# Spatiotemporal Transcriptome Analysis Reveals Activation of the AP1 Pathway in the Ovarian Microenvironment during the Transition from Premenopause to Postmenopause

**DOI:** 10.14336/AD.2023.0707-1

**Published:** 2024-04-01

**Authors:** Wendi Pei, Xiaomin Cao, Puyao Zhang, Lin Fu, Ping Zhou, Ye Liu, Yong Fan, Fengqin Xu, Canhui Cao, Yang Yu

**Affiliations:** ^1^Center for Reproductive Medicine, Department of Obstetrics and Gynecology, Beijing Key Laboratory of Reproductive Endocrinology and Assisted Reproductive Technology and Key Laboratory of Assisted Reproduction, Ministry of Education, Peking University Third Hospital, Beijing, China.; ^2^Reproductive Medicine Center, Tianjin First Central Hospital, Tianjin, China.; ^3^Key Laboratory for Major Obstetric Diseases of Guangdong Province, The Third Affiliated Hospital of Guangzhou Medical University, Guangzhou, China.; ^4^Department of Gynaecologic Oncology, Key Laboratory of the Ministry of Education, Tongji Hospital, Tongji Medical College, Huazhong University of Science and Technology, Wuhan, China.; ^5^Clinical Stem Cell Research Center, Peking University Third Hospital, Beijing, China

**Dear Editor**,

Although life expectancy in developed countries has increased markedly from 45 to 85 years over the past two centuries, the age at natural menopause (ANM) of healthy women has remained relatively stable (average 50.5, 47-53 years) [1]. Thus, there can be a substantial gap between reproductive ageing and somatic ageing. Factors other than overall lifespan, such as genetics, play a significant role in determining ANM [1, 2]. It is well known that menopause is the end of a woman’s reproductive period and menstrual cycles. However, the molecular basis of the transition from premenopause to postmenopause in the ovaries remains to be determined, especially with respect to the changes in gene expression and cell type components. Herein, we performed spatial transcriptomic sequencing of human pre- and postmenopausal ovaries (n=4, 32-56 years), linked with single-cell transcriptomic analysis of ovaries from young (n=4, 4-5 years) and old (n=4, 18-20 years) nonhuman primates (NHPs) [3], to characterize the changes in expression in human ovaries from premenopause to postmenopause with spatial and single-cell resolution (Fig. 1A).

**Figure 1.**
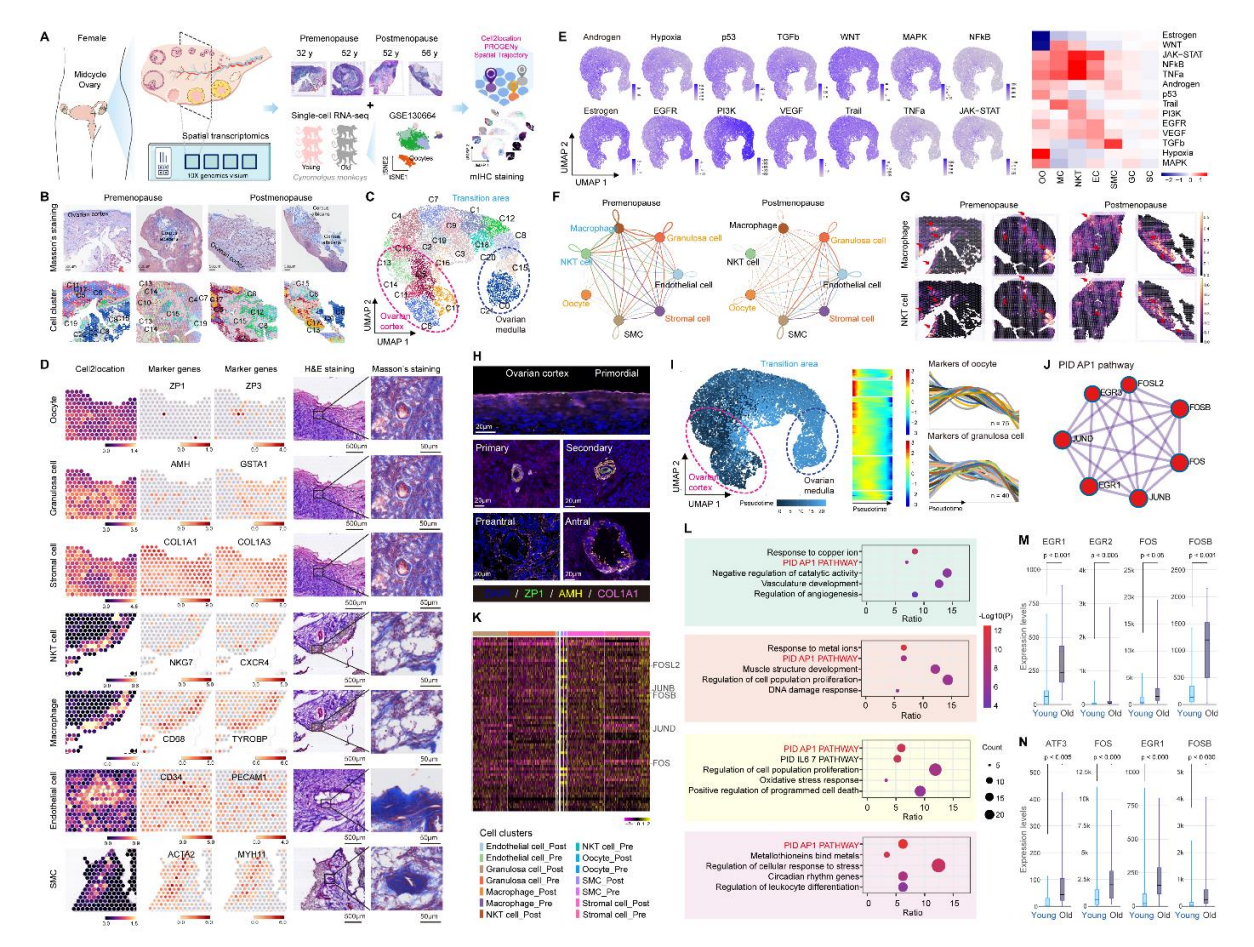
**Spatiotemporal characteristics of human ovaries during the transition from premenopause to postmenopause**. **(A)** Study flowchart of sequencing and analysis. **(B)** Masson’s staining of samples and spot cluster spatial transcriptomics data via Cell2location. **(C)** UMAP plot of spatial transcriptomic spots. Each colour represents a cluster. **(D)** Spatial transcriptomic spot cell type predictions, marker gene expression, H&E staining, and Masson’s staining of the corresponding area. The scale colour of Cell2location analysis establishes the spatial mapping of the cell types. **(E)** PROGENy results of signalling pathways in the UMAP plot and cell types. **(F)** Cell communication analysis among cell types via CellPhoneDB. The nodes represent cell types and are distinguished by different colours. Each line between nodes represents the relationship of cell type A with cell type B. The node with the same colour as the line represents the beginning, and the other node represents the end. The thickness of the line represents the number of ligand-receptor pairs. **(G)** Immune cell distribution at spatial resolution via Cell2location analysis. **(H)** fm-IHC results of the different stages of follicles and stromal cells. **(I)** Pseudotime trajectory analysis of all spatial spots by Monocle2. The UMPA plot of spatial spots is coloured by pseudotime. The heatmap shows the gene expression patterns in each cluster along the pseudotime trajectory, coloured by gene expression levels. The colour intensity indicates the average gene expression. Expression changes in marker genes of oocytes and granulosa cells across the pseudotime trajectory are shown in the scatter plot. **(J)** Main components of the AP1 pathway. **(K)** Heatmap of the cell types in the ovaries between premenopause and postmenopause. **(L)** Representative pathway and process enrichment terms for upregulated genes of postmenopause vs. premenopause in stromal cells, SMCs, NKT cells and macrophages (from top to bottom). AP1 pathway-associated genes upregulated in macrophages (M) and NKT cells (N) in old NHP ovaries at the single-cell transcriptomic level.

We then used the PROGENy algorithm [5] and found that signalling pathways were activated in different structures or cell types, such as the PI3K pathway in the ovarian medulla and the oestrogen pathway in postmenopausal ovaries and granulosa cells (Fig. 1E, Supplementary Fig. 1E-G). Altered intercellular communication is one of the hallmarks of human ageing [6]. In this study, the total number and overall strength of intercellular interactions of cell types at spatial levels were reduced in postmenopausal samples (Fig. 1F). Of note, cell interactions between stromal cells and granulosa/immune cells were significantly decreased (Supplementary Fig. 2A-F). To explore potential mechanisms underlying the changes, we compared the cell distribution and found that macrophages colocalized with NKT cells (Fig. 1G, Supplementary Fig. 3A-B), with higher abundances at premenopause (Supplementary Fig. 3C-E), which was the same as the trend observed in NHP and mouse ovaries (Supplementary Fig. 3F-H). For stromal cells, strong colocalization signals with oocytes and granulosa cells were found and validated (Fig. 1H, Supplementary Fig. 3A-B), and the expression levels increased at premenopause (Supplementary Fig. 4A-B). In contrast, the expression levels of SMCs were decreased at premenopause (Supplementary Fig. 4A), indicating stronger angiogenesis after menopause. The differential activation patterns of stromal cells and SMCs were validated in human ovaries and were also identified in NHP ovaries (Supplementary Fig. 4C-E).

The ovaries are the primary sources of oestrogen in a woman's body, and the decline in their function during the transition to menopause leads to the end of menstruation and fertility [3]. A previous study revealed dynamic changes in mechanical components and showed that collagen expression in the ovaries peaks at reproductive age [7], which is consistent with the results of this study. Indeed, the localization of follicles in the collagen-rich cortex provides an environment that supports follicle architecture and probably plays a role in follicle survival [8], while ovarian stiffness or fibrosis limits oocyte maturation and is associated with ovarian ageing [9]. Additionally, as follicles grow and ovulate and corpora lutea develop, cyclic structural changes may also affect the distribution of stromal cells [10]. However, the mechanisms of dynamic changes in the collagen component of stromal cells during the ovary transition should be further determined. AP1 is a key transcription factor regulating several cellular processes associated with cell survival proliferation and differentiation, and dysregulated expression and activity of AP1 have been implicated in several severe diseases, especially inflammatory disorders and cancer [11]. Research on the role of the AP1 pathway in ovarian development and ageing is currently limited. However, existing evidence indicates that in human ovaries, the expression of AP1 subunits is elevated in periovulatory follicles and is regulated by human chorionic gonadotropin [12]. Further studies are needed to explore the specific mechanisms by which the AP1 pathway influences ovarian function and the potential role of this pathway in ovarian ageing.

Due to the rarity of ovarian samples, the sample size of this study was small, which may have caused bias in the results and conclusions. In addition, although humans and NHPs share a high degree of genetic similarity, there are differences in gene expression between the two species. Therefore, future studies with a larger sample size and analyses of the differences between species regarding ovary ageing are needed.

Overall, this study provides a spatiotemporal perspective for clarifying the changes in the function of human ovaries during the transition from premenopause to postmenopause. During the transition, a significant shift occurs in gene expression and intercellular interactions among different cell types. Notably, the AP1 pathway becomes activated within the ovarian microenvironment. In this study, the spatial distribution of spots and clusters exhibited distinct structural characteristics, wherein the gene expression profiles along the pseudotime trajectory were enriched in processes associated with hormones or stress responses as well as the AP1 pathway. Taken together, these findings provide insights into the complex biological transition of ovary ageing with spatial and single-cell resolution.

## Supplementary Materials

The Supplementary data can be found online at: www.aginganddisease.org/EN/10.14336/AD.2023.0707-1.


