# Supplementary material for: Spatiotemporal Transcriptome Analysis Reveals Activation of the AP1 Pathway in the Ovarian Microenvironment during the Transition from Premenopause to Postmenopause
Source: Aging Dis. 2024 Apr 1;15(2):445–8. doi: 10.14336/AD.2023.0707-1 (PMC10917534; doi:10.14336/AD.2023.0707-1)
Supplement: Supplementary file 1 [file AD-15-2-445-s.pdf]

SUPPLEMENTARY DATA

Spatiotemporal Transcriptome Analysis Reveals Activation of the AP1 Pathway in the Ovarian Microenvironment during the Transition from Premenopause to Postmenopause

Wendi Pei, Xiaomin Cao, Puyao Zhang, Lin Fu, Ping Zhou, Ye Liu, Yong Fan, Fengqin Xu, Canhui Cao, Yang Yu

© 2023. Pei W et al. Published online at http://www.aginganddisease.org/EN/10.14336/AD.2023.0707-1

SUPPLEMENTARY DATA

MATERIALS AND METHODS

Sample preparation and ethical statement

The sample collection was approved by the Research Ethics Committee of the Peking University Third Hospital (IRB00006761-M2022616) and was conducted following approved institutional guidelines. The donated human ovaries were obtained from women who had undergone oophorectomy due to benign lesions. The samples were cut into 6 x 6 x 5 mm pieces and dried with lab blotting paper to prevent ice crystal formation. Subsequently, we embedded the tissue in OCT in the embedding box in dry ice and stored it at -80°C.

Spatial RNA-seq on the 10× Genomics platform

Spatial transcriptomics analysis was carried out using the 10x Genomics Visium platform [1]. Ten micrometre tissue sections from OCT-embedded fresh frozen human ovaries were mounted onto Visium Spatial slides and permeabilized for 30 min to release mRNAs, which bound to the spatially barcoded oligos present in the underlying spots and were reverse transcribed according to the manufacturer's protocol. Libraries prepared from the cDNAs were sequenced on the Illumina NovaSeq 6000 platform at >50,000 reads per spot, generating >400 M reads per section.

Single-cell RNA-seq analysis

Single-cell RNA-seq data of young (n = 4) and aged (n = 4) nonhuman primates, which were generated using the STRT-seq protocol, were downloaded from GSE130664 [2]. In total, 2,601 cells from primate ovaries were processed by Seurat, with the following criteria: (1) log2 (the mean expression of the cluster (transcripts per kilobase million [TPM]) greater than 0.5, (2) power value greater than 0.25, and (3) percentage of cluster cells (PCT.1) greater than 0.3.

Spatial RNA-seq analysis

Space Ranger software (version 1.3.0, 10x Genomics) was used to align and obtain raw counts from each of the spots on the Visium spatial transcriptomics slides against the GRCh38 human genome reference data. The Space Ranger output files were then imported into the R environment (V4.0.5) and analysed using the R package Seurat v4.0.1 (https://github.com/satijalab/seurat/). We obtained information about the number of spots, UMIs under each tissue, median/mean genes and reads per spot, etc. SCTransform was used to normalize the data, followed by PCA. The spatial expression of selected genes was visualized by using the normalized data.

Cell-type annotation of the spatial transcriptomic data

Cell2location [3] performs deconvolution in two steps, i.e., training the regression model and building the cell2location model. In training the regression model, the function "filter_genes" was used to filter the genes based on default parameters. Then, the function "cell2location.models. RegressionModel.setup_anndata" with default parameters was used to build an annData object. After applying the function "RegressionModel" to create a regression model, the function "mod.train" with the parameters "max_ epochs = 1000, use_gpu=False" was used to train the regression model. Based on the trained model, functions "adata_scrna_raw. varm" and "adata_scrna_raw.var" were implemented to export the estimated expression in each cell subpopulation. The function "np.intersect1d" was used to find shared genes and subset both the spatial Visium data and reference signatures. For the model construction, the function "cell2location.models.Cell2location.setup_anndata" was used to prepare the anndata to construct the cell2location model. Functions "cell2location.models. Cell2location" and "mod.train" were used to create and train this model. Finally, the function "plot_spatial" was used to visualize cell abundance in spatial coordinates.

Analysis of DEGs and GO and pathway enrichment analysis

We used the FindMarkers function to perform differential gene expression analysis between regions and set p_val_adj = 0.05 as the cut-off value. DEGs were visualized as a heatmap with the R package ComplexHeatmap. GO analysis of these DEGs was performed by Metascape (version 3.5, http://metascape.org/), and selected pathway terms are displayed in the heatmap.

Spatial trajectory analysis

The pseudotime trajectories of all spatial spots were generated with the Monocle2 package in R4.0.3. The newCellDataSet(), estimateSizeFactors(), and estimateDispersions() were used to perform these analyses. DetectGenes() was used to filter low-quality cells with "min_expr = 0.1".

© 2023. Pei W et al. Published online at http://www.aginganddisease.org/EN/10.14336/AD.2023.0707-1

SUPPLEMENTARY DATA

PROGENy analysis

We used a footprint-based method called PROGENy (Pathway RespOnsive GENes for activity inference) [4] to estimate signalling pathway activities based on consensus gene signatures obtained from perturbation experiments. PROGENy contains signatures for 14 signalling pathways (Androgen, EGFR, Estrogen, Hypoxia, JAK-STAT, MAPK, NFkB, p53, PI3K, TGFb, TNFa, Trail, VEGF, and WNT). In data preprocessing, Seurat function "SCTransform" was used to normalize spatial data. Then, the SCT assay data was used as input to the function "progeny" of PROGENy. The pathway activity score was calculated with the function progeny from the PROGENy R package v1.16.0 using the 500 most responsive genes per pathway. Average pathway activity scores for different cell types or samples were used to assess the differences. Heatmaps were generated using the R package PHEATMAP v1.0.12.

Cell communication analysis

Raw sequence reads in FASTQ format from ovaries were processed and aligned to the human reference transcriptome (https://www.10xgenomics.com/) using the Cellranger v4.0.0 pipeline with the default settings. The cell types were annotated by the Cell2location according to the single-cell transcriptomic data of GSE130664. The interactions between cell types identified in ovary samples were analysed by CellPhoneDB v.3.0 (https://github.com/ventolab/CellphoneDB) [5,6]. We retrieved the interacting pairs of ligands and receptors satisfying the following criteria: all members were expressed in at least 10% of the cells in the cluster under consideration. We used iTALK (https://github.com/Coolgenome/iTALK) and igraph software for cellphoneDB results to demonstrate the cell type interaction networks. The shell diagram is a split presentation of the network diagram.

Histological evaluation and immunohistochemistry

For histological examination, the indicated tissues were harvested, fixed with 4% paraformaldehyde in PBS, embedded into paraffin blocks, sectioned and then stained with H&E following standard protocols. For immunohistochemistry (IHC) analysis, deparaffinized sections were incubated with primary antibodies at 4°C overnight and then incubated with species-appropriate secondary antibodies (1:200, Servicebio). The antibodies used are listed in the Supplementary Table 5. The images were taken with an inverted microscope.

Immunofluorescence

After deparaffinization and rehydration, sections were subjected to antigen retrieval using citrate antigen repair buffer (pH 6.0) or EDTA antigen repair buffer (pH 9.0) at 100°C for 20 min, blocked with 3% BSA for 30 min at room temperature, and incubated in primary antibodies at 4°C overnight. Then, the sections were incubated with the corresponding secondary antibodies (1:200 dilution) for 1 h at room temperature. Polychromatic immunofluorescence staining for CD3, CD68, ZP1, and ZP3 was performed with the tyramide signal amplification (TSA) technique. In brief, tissue sections were incubated with primary antibodies as described in the above IHC protocol in two sequential cycles before the application of corresponding secondary antibodies and TSA solution for iF488 (G1231, Servicebio) and CY3 (G1223, Servicebio). After the last TSA cycle, DAPI was counterstained for 10 min. Fluorescent images were obtained with an automatic digital slide scanner (Pannoramic MIDI). The mean fluorescence intensities were measured using ImageJ software. Antibody information is listed in the Supplementary Table 5.

Masson's staining

The ovary sections were brought to room temperature and incubated in potassium dichromate overnight. After a wash in distilled water, the sections were stained in iron haematoxylin working solution for 1 min and rinsed with running tap water. Then, the slides were stained in Ponceau-acid fuchsin solution for 6 min. After a wash with running tap water, the sections were differentiated in the phosphomolybdic acid solution for 1 min and directly transferred to an aniline blue solution for 30 s. Next, the sections were briefly rinsed in distilled water, followed by differentiation in 1% acetic acid solution for 2-5 min. Finally, the slides were dehydrated in absolute ethyl alcohol, mounted with resinous mounting medium, and examined by microscopy.

Statistical analyses

Data are presented as the mean ± SD. All experimental data were analysed using an unpaired two-sided Student's t test or one-way analysis of variance (ANOVA) to assess differences between groups (GraphPad 7.0 Software). *p value < 0.05 was considered to indicate statistical significance.

© 2023. Pei W et al. Published online at http://www.aginganddisease.org/EN/10.14336/AD.2023.0707-1

SUPPLEMENTARY DATA

Ethics approval

The human sample collection was approved by the Research Ethics Committee of the Peking University Third Hospital (IRB00006761-M2022616) and was conducted following approved institutional guidelines. The animal experiments were approved by the Animal Experiment Ethics Committee of Peking University Third Hospital (A2022001) and performed according to the AVMA guidelines.

Data availability statements

Spatial transcriptomic sequencing of human pre/postmenopausal ovaries is available at the Genome Sequence Archive (HRA004049). Single-cell RNA-seq data were downloaded from GSE130664. Cell clusters of single-cell transcriptomic data could be found at the website, https://doi.org/10.7910/DVN/D8RZKK. Any additional information is available from the lead contact upon request.

References

[1] Fawkner-Corbett D, Antanaviciute A, Parikh K, Jagielowicz M, Gerós AS, Gupta T, et al (2021). Spatiotemporal analysis of human intestinal development at single-cell resolution. Cell,184(3):810-826.

[2] Wang S, Zheng Y, Li J, Yu Y, Zhang W, Song M, et al (2020). Single-Cell Transcriptomic Atlas of Primate Ovarian Aging. Cell, 180(3):585-600 e519.

[3] Kleshchevnikov V, Shmatko A, Dann E, Aivazidis A, King HW, Li T, et al (2022). Cell2location maps fine-grained cell types in spatial transcriptomics. Nat Biotechnol, 40(5):661-671.

[4] Schubert M, Klinger B, Klünemann M, Sieber A, Uhlitz F, Sauer S, et al (2018). Perturbation-response genes reveal signaling footprints in cancer gene expression. Nat Commun, 9(1):20.

[5] Efremova M, Vento-Tormo M, Teichmann SA, Vento-Tormo R (2020). CellPhoneDB: inferring cell-cell communication from combined expression of multi-subunit ligand-receptor complexes. Nat Protoc, 15(4):1484-1506.

[6] Garcia-Alonso L, Handfield LF, Roberts K, Nikolakopoulou K, Fernando RC, Gardner L, et al (2021). Mapping the temporal and spatial dynamics of the human endometrium in vivo and in vitro. Nat Genet, 53(12):1698-1711.

© 2023. Pei W et al. Published online at http://www.aginganddisease.org/EN/10.14336/AD.2023.0707-1

SUPPLEMENTARY DATA

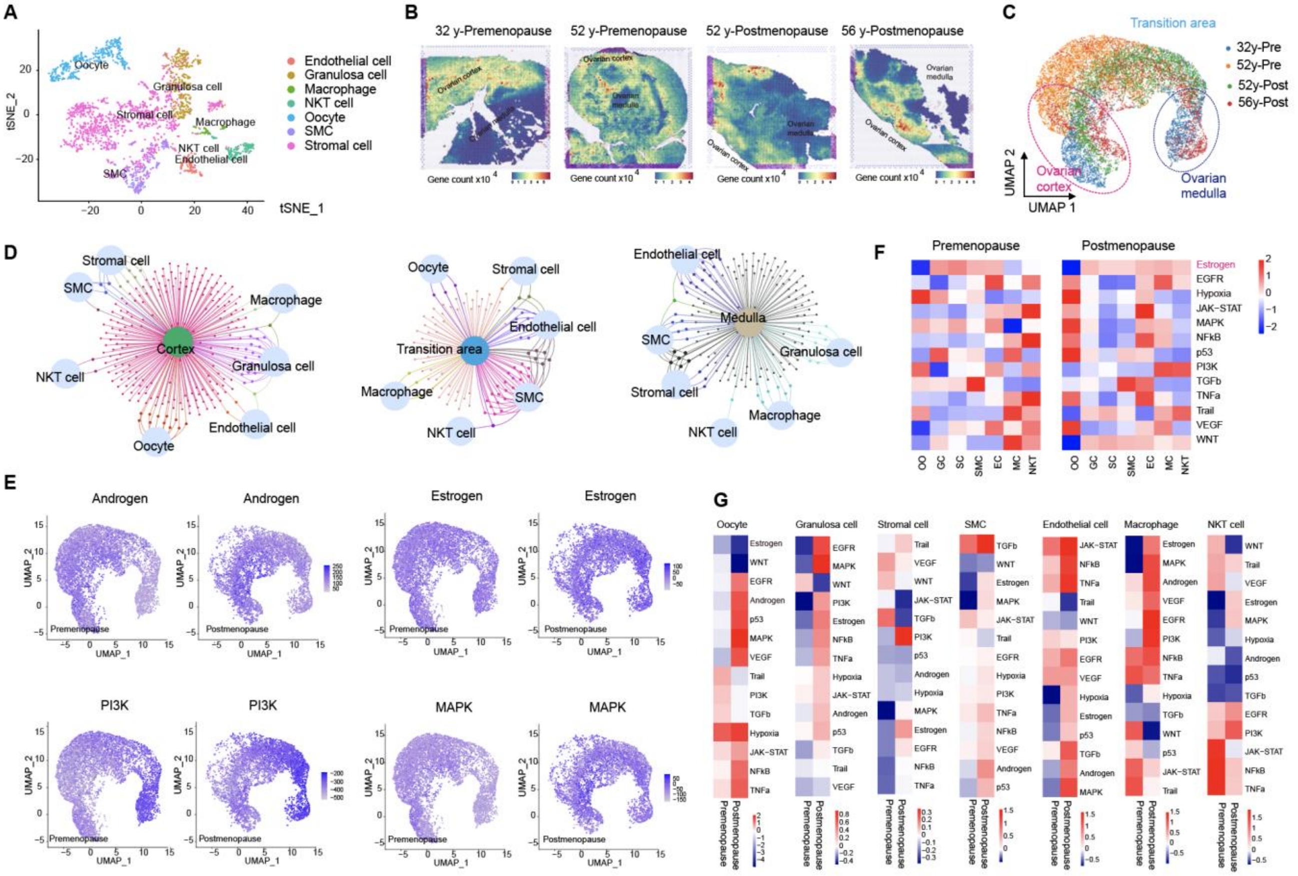

Supplementary Figure 1. Spatial transcriptomic characteristics of samples and PROGENy analysis of cell types. (**A**) Single-cell transcriptomic analysis of NHP ovaries from GSE130664. Seven types of cell clusters are indicated, and each colour represents a cell cluster. (**B**) Gene expression levels of spatial spots in different samples. (**C**) UMAP plot of spatial spots of samples. Each colour represents a sample. (**D**) Venn network analysis of differentially expressed genes (DEGs) of the ovarian cortex, transition area, and medulla and marker genes of each cell type. (**E**) PROGENy analysis of the androgen, estrogen, PI3K, and MAPK pathways between premenopause and postmenopause. (**F**) PROGENy analysis of different pathways in premenopause and postmenopause. (**G**) PROGENy analysis of different pathways in different cell clusters between premenopause and postmenopause.

SUPPLEMENTARY DATA

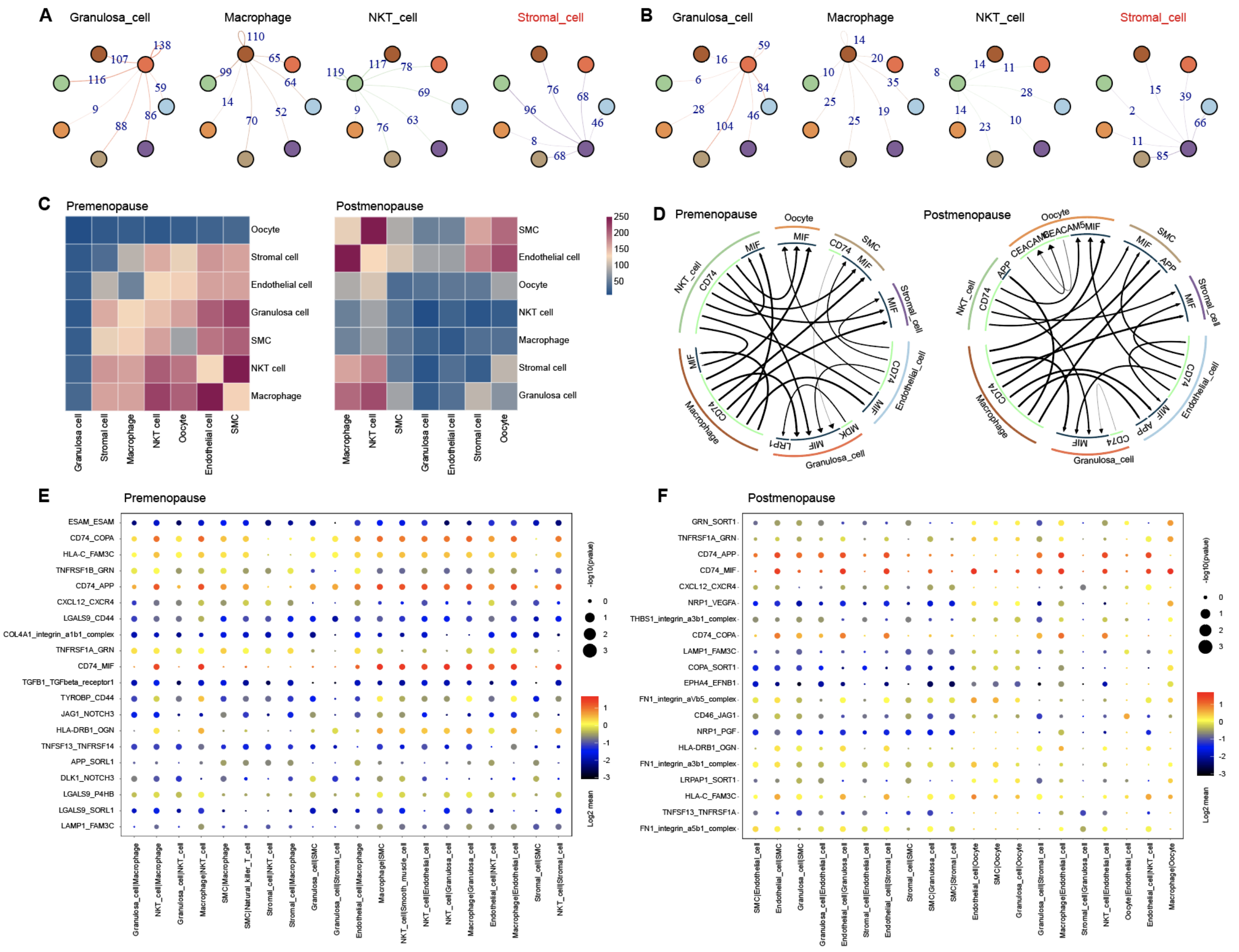

Supplementary Figure 2. Intercellular communication analysis of the cell clusters. Communications among cell types in premenopausal samples (**A**) and postmenopausal samples (**B**) at the spatial transcriptomic level. We used a shell diagram to show the communication networks of each cell type. Each line represents the relationship from cell type A to cell type B. The number represents ligand–receptor pairs. The higher the number, the thicker the line. (**C**) Heatmaps of communication among cell types in pre- and postmenopausal samples. (**D**) The top ligand–receptor networks of cell types in pre- and postmenopausal samples. The arrow represents the pointing relationship, the thickness of the line represents the expression of the departure gene, and the size of the arrow represents the expression of the gene in the accepting direction. The light green represents the departure direction, and the dark green represents the acceptance direction. If a protein/complex is both a ligand and an acceptor, it is light green. Dot plot of the top ligand–receptor pairs among cell types in premenopausal samples (**E**) and postmenopausal samples (**F**).

© 2023. Pei W et al. Published online at http://www.aginganddisease.org/EN/10.14336/AD.2023.0707-1

SUPPLEMENTARY DATA

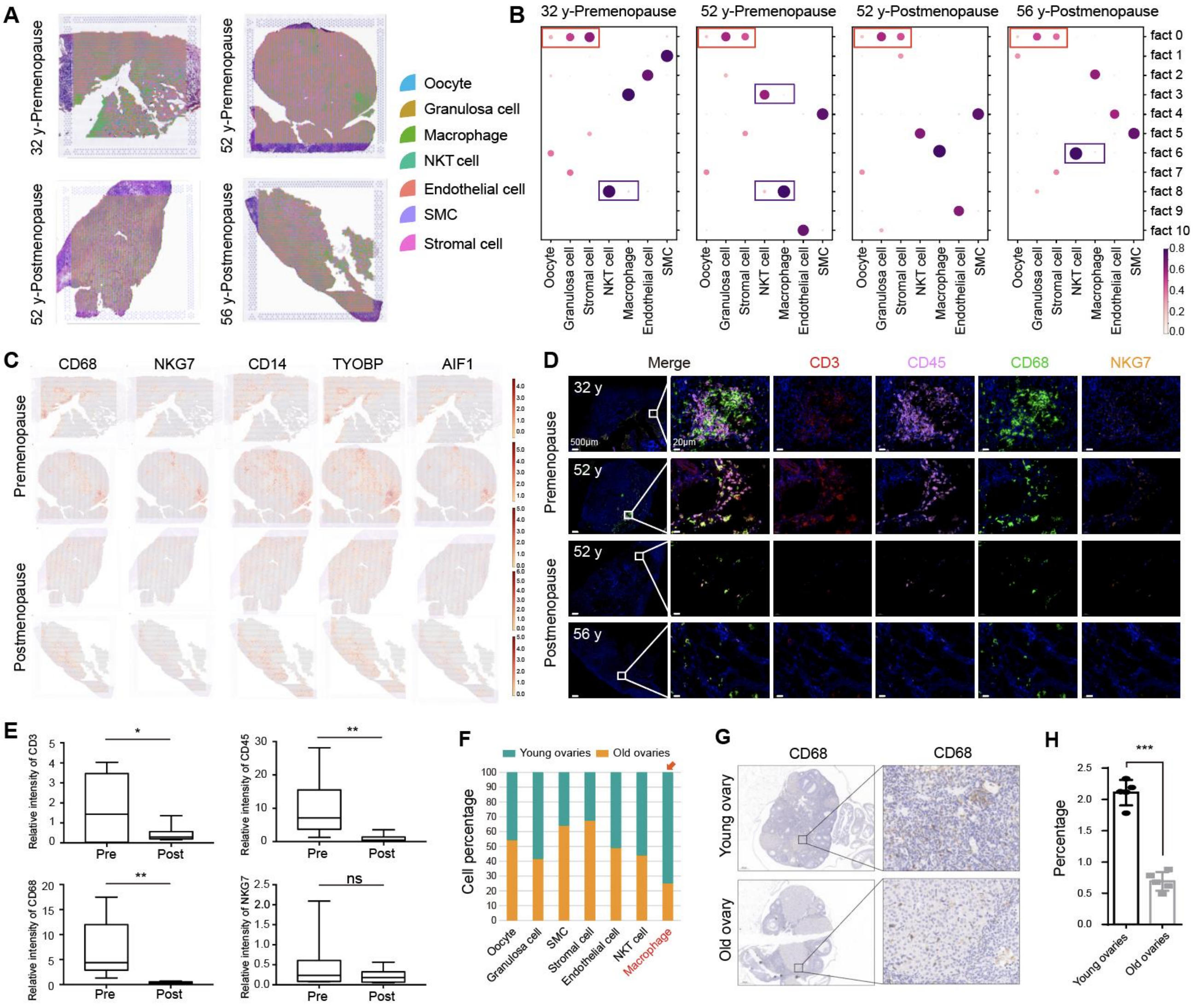

Supplementary Figure 3. The expression features of immune cells in human, NHP and mouse ovaries. (**A**) Using the single-cell RNA-seq data, we performed factor analysis to determine the likely single-cell composition of each spot via Cell2location. (**B**) Nonnegative matrix factorization (NMF) weight analysis among the 7 cell types in the spatial transcriptomic spots. Dot colour shade and size indicate the weight obtained for each cell type evaluated in each NMF component. (**C**) The expression levels of immune cell markers at the spatial transcriptomic level. (**D**) fm-IHC staining of immune cells among samples. (**E**) Statistical analysis of immune cell markers between pre/postmenopausal samples. (**F**) Cell proportion analysis of cell types between young and old ovaries from the single-cell dataset GSE130664. IHC staining (**G**) and statistical analysis (**H**) of Cd68 between young and old mouse ovaries.

© 2023. Pei W et al. Published online at http://www.aginganddisease.org/EN/10.14336/AD.2023.0707-1

SUPPLEMENTARY DATA

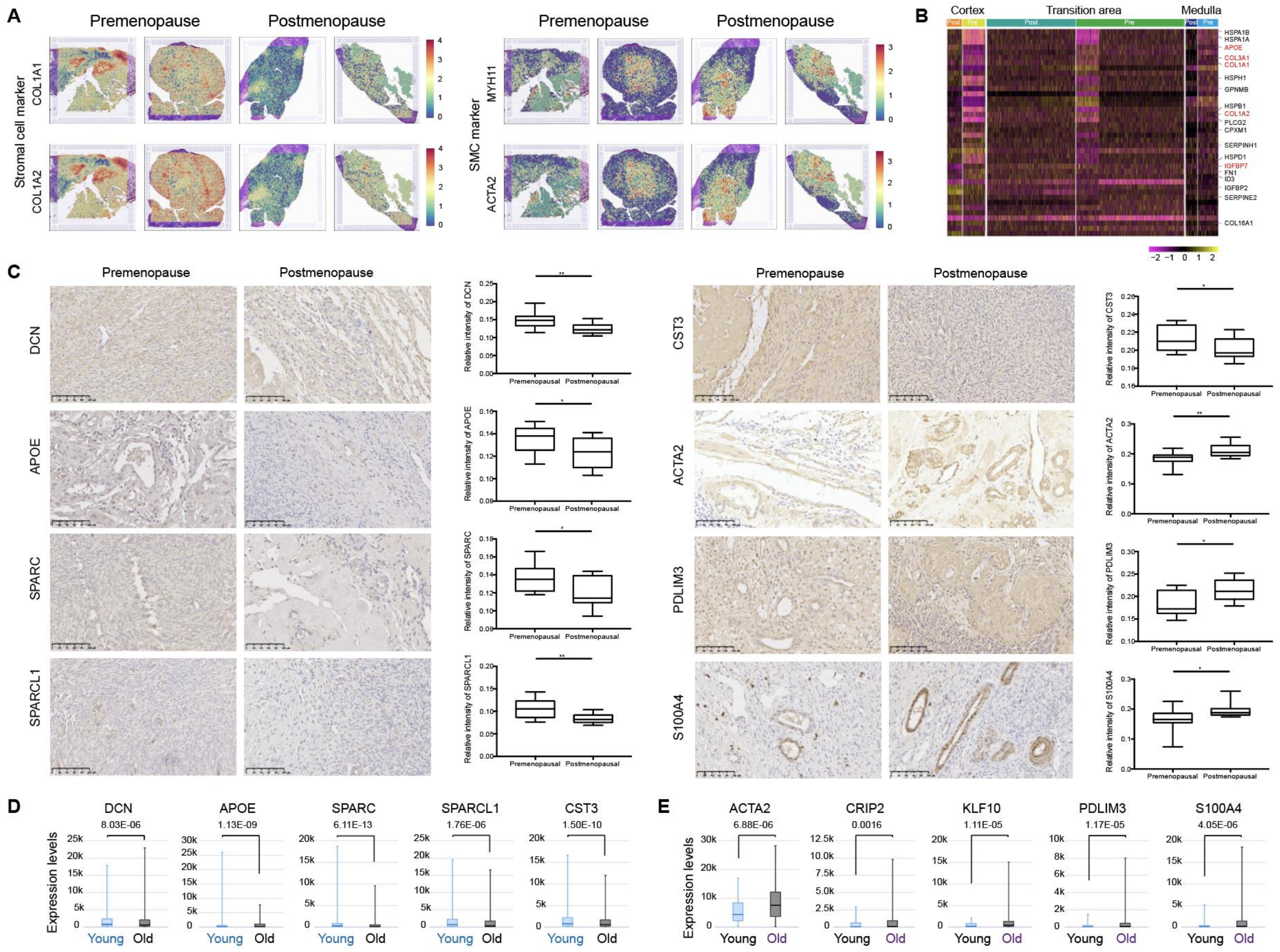

Supplementary Figure 4. Expression patterns of stromal cells and SMCs in the human and NHP ovaries. (**A**) The expression levels of immune cell markers at the spatial transcriptomic level. (**B**) Heatmap of the cortex, transition, and medulla area in human ovaries between premenopause and postmenopause. (**C**) IHC staining and statistical analysis of makers of stromal cells and SMCs between premenopause and postmenopause. The genes upregulated in stromal cells in young NHP ovaries (**D**) and the genes upregulated in SMCs in old NHP ovaries (**E**) at the single-cell transcriptomic level.

© 2023. Pei W et al. Published online at http://www.aginganddisease.org/EN/10.14336/AD.2023.0707-1

SUPPLEMENTARY DATA

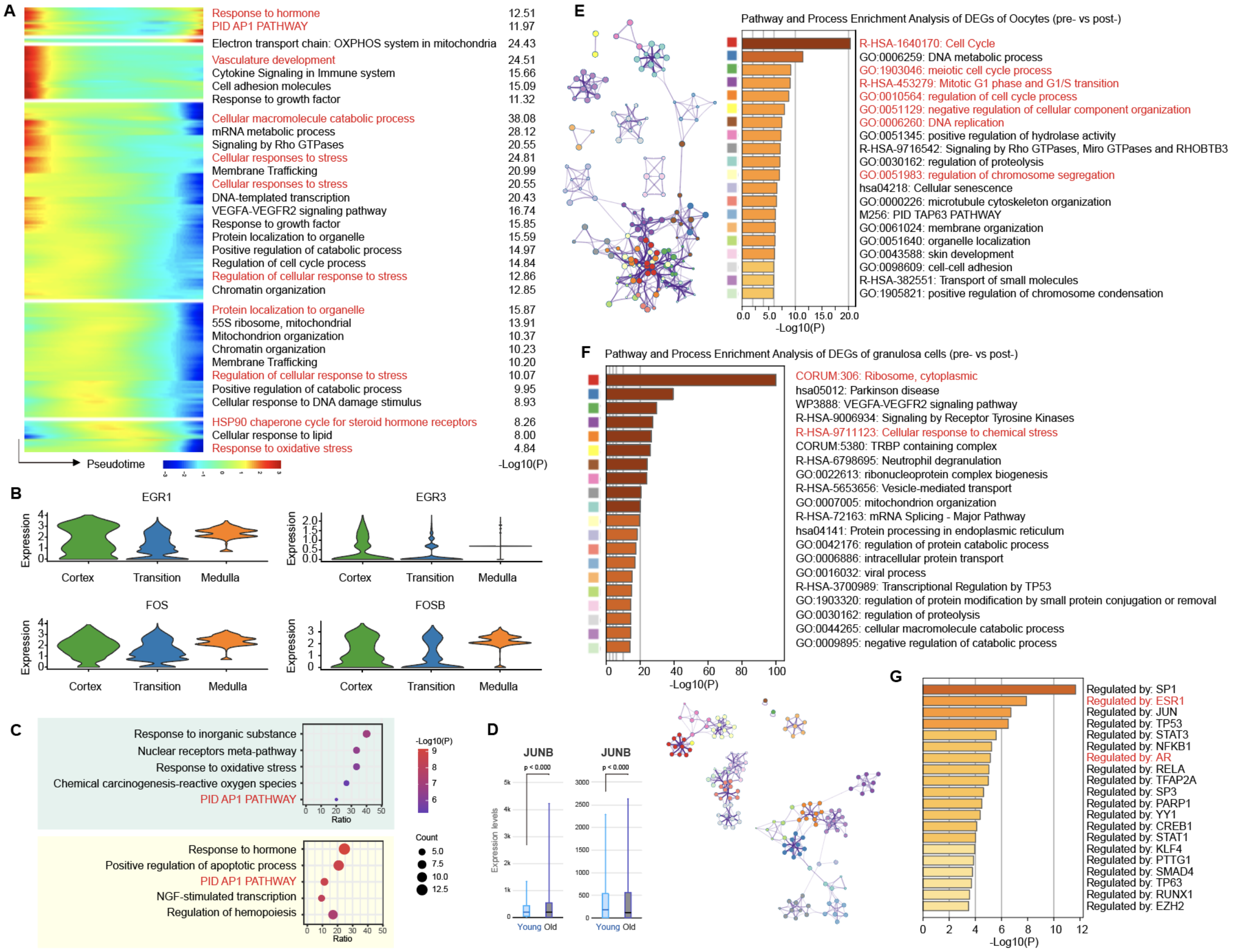

Supplementary Figure 5, Pseudotime trajectory analysis, AP1 pathway analysis, and enrichment analysis of DEGs of oocytes and granulosa cells. (**A**) Heatmap showing the dynamic DEGs along the pseudotime trajectory. According to the cluster analysis, the distribution of DEGs was divided into 6 clusters. The processes enriched in each subtype are listed on the right. (**B**) The different expression patterns of AP1 pathway-associated genes along the pseudotime trajectory. (**C**) Representative pathway and process enrichment terms for upregulated genes of old NHP ovaries in stromal cell (top) and NKT cell (bottom). (**D**) The AP1 pathway-associated genes upregulated in SMCs (left) and stromal cells (right) in old NHP ovaries at the single-cell transcriptomic level. Pathway and process enrichment analysis of the DEGs of oocytes (**E**) and granulosa cells (**F**) between premenopause and postmenopause. (**G**) Transcription factors analysis in TRRUST (v2.0) of DEGs in granulosa cells between premenopause and postmenopause.

© 2023. Pei W et al. Published online at http://www.aginganddisease.org/EN/10.14336/AD.2023.0707-1

SUPPLEMENTARY DATA

Supplementary Table 1. Quality control analysis of spatial transcriptomic data.

Summary	32y-Pre	52y-Pre	52y-Post	56y-Post
**Number of Reads**	245,284,460	205,352,644	243,576,742	212,291,518
**Number of Spots Under Tissue**	3,206	4,242	3,212	2,431
**Fraction Reads in Spots Under Tissue**	91.30%	92.00%	92.70%	90.80%
**Mean Reads per Spot**	76,508	48,409	75,833	87,327
**Mean Reads Under Tissue per Spot**	67,823	43,596	68,477	76,653
**Median UMI Counts per Spot**	8,571	10,503	6,666	7,323
**Median Genes per Spot**	2,868	3,202	2,320	2,467
**Total Genes Detected**	23,470	24,113	22,728	22,459
**Reads Mapped to Genome**	93.10%	95.90%	93.80%	92.20%
**Reads Mapped Confidently to Genome**	90.10%	92.10%	89.20%	87.20%
**Reads Mapped Confidently to Intergenic Regions**	1.80%	1.60%	2.10%	1.80%
**Reads Mapped Confidently to Intronic Regions**	3.20%	3.50%	3.90%	4.10%
**Reads Mapped Confidently to Exonic Regions**	85.10%	87.10%	83.30%	81.30%
**Reads Mapped Confidently to Transcriptome**	83.30%	85.20%	81.50%	79.60%
**Reads Mapped Antisense to Gene**	0.50%	0.60%	0.60%	0.50%

Supplementary Table 2. DEGs of macrophages between the young and old NHP ovaries. Cell clusters of single-cell transcriptomic data. Found at: https:// doi.org/10.7910/DVN/ D8RZKK.

Tissue	Cell Type	Class	Gene	Avg_logFC	P_val
**Ovary**	M	OvsY.up	ADAMTS1	0.778194	0.00066693
**Ovary**	M	OvsY.up	ADH5	1.67125	0.02514208
**Ovary**	M	OvsY.up	AICDA	1.03086	0.04173586
**Ovary**	M	OvsY.up	APOD	2.21561	0.00150051
**Ovary**	M	OvsY.up	AQP11	0.949935	0.00436886
**Ovary**	M	OvsY.up	ARID1B	3.69342	0.01534768
**Ovary**	M	OvsY.up	ARID5B	1.90255	0.00630426
**Ovary**	M	OvsY.up	ARPC4	0.959754	0.00751762
**Ovary**	M	OvsY.up	ATG101	4.49833	0.01395118
**Ovary**	M	OvsY.up	ATP5C1	0.897773	0.0363182
**Ovary**	M	OvsY.up	ATP6V0C	0.558482	0.0305432
**Ovary**	M	OvsY.up	BAZ2B	2.38025	0.00081652
**Ovary**	M	OvsY.up	BCL2L11	0.590356	0.01915052
**Ovary**	M	OvsY.up	BHLHE40	1.9011	0.00766345
**Ovary**	M	OvsY.up	BNIP3	2.88783	0.02349596
**Ovary**	M	OvsY.up	BRI3	2.07367	0.04021393
**Ovary**	M	OvsY.up	C15orf48	2.58014	0.00459349
**Ovary**	M	OvsY.up	C7	1.62252	0.01412396
**Ovary**	M	OvsY.up	CALD1	0.961784	2.21E-06
**Ovary**	M	OvsY.up	CALR	1.04225	0.02970558
**Ovary**	M	OvsY.down	CAPG	-0.76021	0.04498583
**Ovary**	M	OvsY.up	CCL11	0.641224	0.03025845
**Ovary**	M	OvsY.up	CD14	0.56101	0.00908249
**Ovary**	M	OvsY.up	CD44	1.23342	0.04575892
**Ovary**	M	OvsY.up	CDC5L	2.86859	0.03385815
**Ovary**	M	OvsY.up	CFAP36	2.05834	0.02637569
**Ovary**	M	OvsY.up	CHD2	1.12725	0.0151845
**Ovary**	M	OvsY.up	CLU	0.541448	0.00565296
**Ovary**	M	OvsY.up	CNN1	2.02825	0.00517442
**Ovary**	M	OvsY.up	COX17	0.98789	0.04326221
**Ovary**	M	OvsY.up	COX20	1.67633	0.04064835
**Ovary**	M	OvsY.up	COX6B1	1.34332	0.00270915
**Ovary**	M	OvsY.up	COX8A	0.819429	0.00593168
**Ovary**	M	OvsY.up	CRHBP	1.1357	0.02605035
**Ovary**	M	OvsY.up	CSRNP1	0.588363	0.04510988

© 2023. Pei W et al. Published online at http://www.aginganddisease.org/EN/10.14336/AD.2023.0707-1

**Ovary**	M	OvsY.up	CST3	1.01993	0.00050318
**Ovary**	M	OvsY.down	CTSB	-0.86908	0.01671242
**Ovary**	M	OvsY.down	CTSD	-1.13379	0.00773248
**Ovary**	M	OvsY.up	CUTA	2.75957	0.00183615
**Ovary**	M	OvsY.down	CYTB	-1.67742	0.00967385
**Ovary**	M	OvsY.up	DCN	1.18153	0.00948083
**Ovary**	M	OvsY.up	DDAH2	1.70125	0.00399521
**Ovary**	M	OvsY.up	DDRGK1	1.23076	0.00929375
**Ovary**	M	OvsY.up	DPYD	0.847349	0.00387975
**Ovary**	M	OvsY.up	DST	0.517928	0.01582068
**Ovary**	M	OvsY.up	EBNA1BP2	0.958167	0.04138999
**Ovary**	M	OvsY.up	EEF1D	0.717503	9.26E-05
**Ovary**	M	OvsY.up	EEF1G	0.615812	0.02405955
**Ovary**	M	OvsY.up	EFNA1	1.39246	0.00774823
**Ovary**	M	OvsY.up	EGR1	0.843017	2.93E-05
**Ovary**	M	OvsY.up	EGR2	2.41593	0.00425322
**Ovary**	M	OvsY.up	EIF4A3	1.12467	0.0059723
**Ovary**	M	OvsY.up	ENSMFAG00000000366	0.545139	0.00284407
**Ovary**	M	OvsY.up	ENSMFAG00000000594	1.02195	0.00019986
**Ovary**	M	OvsY.up	ENSMFAG00000001185	3.02371	0.01321284
**Ovary**	M	OvsY.up	ENSMFAG00000002766	0.982351	0.00166303
**Ovary**	M	OvsY.up	ENSMFAG00000002778	1.28735	0.00213808
**Ovary**	M	OvsY.up	ENSMFAG00000007994	1.0867	0.00028717
**Ovary**	M	OvsY.up	ENSMFAG00000011609	3.67107	0.00963559
**Ovary**	M	OvsY.up	ENSMFAG00000018170	0.684274	0.02238595
**Ovary**	M	OvsY.up	ENSMFAG00000024955	0.738411	0.04173895
**Ovary**	M	OvsY.up	ENSMFAG00000026096	1.18186	0.01595272
**Ovary**	M	OvsY.up	ENSMFAG00000027717	0.523236	0.00529406
**Ovary**	M	OvsY.up	ENSMFAG00000030096	0.991923	0.00039395
**Ovary**	M	OvsY.up	ENSMFAG00000030228	1.695	0.00315781
**Ovary**	M	OvsY.up	ENSMFAG00000030582	0.906578	0.00701329
**Ovary**	M	OvsY.up	ENSMFAG00000033495	0.901842	0.00223778
**Ovary**	M	OvsY.down	ENSMFAG00000034345	-1.01984	0.02761902
**Ovary**	M	OvsY.up	ENSMFAG00000035459	0.754122	0.02677971
**Ovary**	M	OvsY.up	ENSMFAG00000035836	1.10475	0.00046307
**Ovary**	M	OvsY.up	ENSMFAG00000036749	1.10593	0.01359847
**Ovary**	M	OvsY.up	ENSMFAG00000036985	1.28784	0.02695623
**Ovary**	M	OvsY.up	ENSMFAG00000037878	1.10098	8.96E-05
**Ovary**	M	OvsY.up	ENSMFAG00000039100	0.843533	0.00279888
**Ovary**	M	OvsY.up	ENSMFAG00000039291	3.22417	0.02036549
**Ovary**	M	OvsY.up	ENSMFAG00000040276	1.45959	0.01458812
**Ovary**	M	OvsY.up	ENSMFAG00000040595	1.16887	0.00074705
**Ovary**	M	OvsY.up	ENSMFAG00000041383	0.736064	0.00346151
**Ovary**	M	OvsY.up	ENSMFAG00000042314	1.55437	1.71E-05
**Ovary**	M	OvsY.up	ENSMFAG00000042652	0.593242	0.01446712
**Ovary**	M	OvsY.down	ENSMFAG00000043121	-0.6985	0.01489661
**Ovary**	M	OvsY.up	ENSMFAG00000043412	0.520291	0.01130395
**Ovary**	M	OvsY.up	ENSMFAG00000044548	1.6866	0.03253923
**Ovary**	M	OvsY.down	ENSMFAG00000044694	-1.21426	0.01703951
**Ovary**	M	OvsY.up	ENSMFAG00000045551	1.49954	0.005272
**Ovary**	M	OvsY.up	ENSMFAG00000045648	1.16619	0.00597113
**Ovary**	M	OvsY.up	ENSMFAG00000045819	0.838923	0.01237762
**Ovary**	M	OvsY.down	FCER1G	-1.33846	0.00212333
**Ovary**	M	OvsY.up	FERMT3	1.74686	0.0222265
**Ovary**	M	OvsY.up	FN1	0.644118	0.02001111
**Ovary**	M	OvsY.up	FOS	1.74519	0.0288182
**Ovary**	M	OvsY.up	FOSB	1.5524	0.00032709
**Ovary**	M	OvsY.up	FRMD8	2.92936	0.01755202
**Ovary**	M	OvsY.up	FRZB	1.56709	0.00149809

© 2023. Pei W et al. Published online at http://www.aginganddisease.org/EN/10.14336/AD.2023.0707-1

**Ovary**	M	OvsY.up	G3BP2	1.92084	0.04981849
**Ovary**	M	OvsY.up	GADD45B	1.50788	9.22E-05
**Ovary**	M	OvsY.up	GAPDH	0.519429	0.01567379
**Ovary**	M	OvsY.up	GLUL	1.21836	0.00821632
**Ovary**	M	OvsY.up	GNAS	0.840639	0.03226885
**Ovary**	M	OvsY.up	GOLPH3	0.920164	0.02966904
**Ovary**	M	OvsY.up	GPR183	2.25194	0.00487212
**Ovary**	M	OvsY.up	GRIPAP1	1.73366	0.01099072
**Ovary**	M	OvsY.up	GSTA5	2.11243	0.01149713
**Ovary**	M	OvsY.up	GSTM3	2.5182	0.0114052
**Ovary**	M	OvsY.up	GSTM5	0.892979	0.01331677
**Ovary**	M	OvsY.up	GTF2B	1.31703	0.00804124
**Ovary**	M	OvsY.up	HES1	1.23001	0.04728321
**Ovary**	M	OvsY.up	HIGD2A	2.25502	0.03141508
**Ovary**	M	OvsY.up	HNRNPC	0.749923	0.01394309
**Ovary**	M	OvsY.up	HNRNPH1	1.038	0.00216813
**Ovary**	M	OvsY.up	HSPA9	0.608933	0.02021266
**Ovary**	M	OvsY.up	HSPB1	1.04498	2.26E-05
**Ovary**	M	OvsY.up	ID2	0.962655	0.00130023
**Ovary**	M	OvsY.up	IER3	1.60405	0.03010508
**Ovary**	M	OvsY.up	IFNGR2	0.744807	0.0169405
**Ovary**	M	OvsY.up	IFRD1	0.757958	0.00545007
**Ovary**	M	OvsY.up	IL13RA1	0.624494	0.00728684
**Ovary**	M	OvsY.up	IRF2BP2	1.933	0.00900651
**Ovary**	M	OvsY.up	ITGAM	1.39838	0.01406867
**Ovary**	M	OvsY.up	KDM6B	1.09782	0.00808602
**Ovary**	M	OvsY.up	LIPN	0.754295	0.03095927
**Ovary**	M	OvsY.up	LITAF	0.524994	0.0031625
**Ovary**	M	OvsY.up	LMAN1	1.63686	0.00273481
**Ovary**	M	OvsY.up	LMCD1	1.97803	0.00053432
**Ovary**	M	OvsY.up	LYZ	1.09563	0.02945215
**Ovary**	M	OvsY.up	MALAT1	1.64654	0.00243826
**Ovary**	M	OvsY.up	MAP2K3	1.05551	0.03613875
**Ovary**	M	OvsY.up	MDK	0.613626	0.01639406
**Ovary**	M	OvsY.up	METAP2	2.42442	0.00822955
**Ovary**	M	OvsY.down	MS4A7	-1.02304	0.04431127
**Ovary**	M	OvsY.up	MTHFD2	2.115	0.00074357
**Ovary**	M	OvsY.up	MYL9	2.44107	6.90E-05
**Ovary**	M	OvsY.up	MYLK	1.30981	0.01743301
**Ovary**	M	OvsY.up	NASP	2.2446	0.04006268
**Ovary**	M	OvsY.up	NDUFS5	1.03626	0.02064821
**Ovary**	M	OvsY.up	NEIL3	0.941497	0.00395334
**Ovary**	M	OvsY.up	NEK2	3.01423	0.01461861
**Ovary**	M	OvsY.up	NFE2L2	1.98423	0.01930207
**Ovary**	M	OvsY.up	NFKBIA	0.578516	0.00010408
**Ovary**	M	OvsY.up	NIPBL	1.62478	0.0144073
**Ovary**	M	OvsY.up	NKTR	0.888633	0.02533087
**Ovary**	M	OvsY.up	PCBP2	1.52718	0.00970698
**Ovary**	M	OvsY.up	PEG3	0.878794	0.03980873
**Ovary**	M	OvsY.up	PHPT1	0.979232	0.02513146
**Ovary**	M	OvsY.up	PIM3	1.86459	0.00189737
**Ovary**	M	OvsY.up	PKN2	2.07836	0.02193272
**Ovary**	M	OvsY.up	PLD3	2.60373	0.00545393
**Ovary**	M	OvsY.up	PNPLA8	1.23179	0.00497023
**Ovary**	M	OvsY.up	POLR2E	0.686783	0.02593443
**Ovary**	M	OvsY.up	PPAP2C	1.23938	0.00828414
**Ovary**	M	OvsY.up	PPDPF	0.95692	0.04784275
**Ovary**	M	OvsY.up	PPIA	0.907847	0.00558788
**Ovary**	M	OvsY.up	PPP1R14A	1.75656	0.00068789

© 2023. Pei W et al. Published online at http://www.aginganddisease.org/EN/10.14336/AD.2023.0707-1

**Ovary**	M	OvsY.up	PRDX1	1.2054	0.04351836
**Ovary**	M	OvsY.up	PRDX4	0.598569	0.00816558
**Ovary**	M	OvsY.up	PSMD4	2.00156	0.00775036
**Ovary**	M	OvsY.up	PTP4A1	0.762515	0.01945902
**Ovary**	M	OvsY.up	PYURF	1.14464	0.02057917
**Ovary**	M	OvsY.up	RAB33A	1.09008	0.00517286
**Ovary**	M	OvsY.up	RAC1	0.934717	0.01924061
**Ovary**	M	OvsY.up	RACK1	0.996933	0.01714369
**Ovary**	M	OvsY.up	RAD21	0.618381	0.00308785
**Ovary**	M	OvsY.down	RAMP2	-1.87502	0.04027059
**Ovary**	M	OvsY.up	REXO2	2.84786	0.00615662
**Ovary**	M	OvsY.up	RPL24	0.742032	0.00014464
**Ovary**	M	OvsY.up	RPL41	0.939276	6.71E-05
**Ovary**	M	OvsY.up	RPL7A	0.74315	0.00078786
**Ovary**	M	OvsY.up	RPLP2	1.01998	0.00118842
**Ovary**	M	OvsY.up	RPS2	1.04209	0.0141093
**Ovary**	M	OvsY.up	RPS23	0.6177	0.04755735
**Ovary**	M	OvsY.up	RPS24	0.617578	0.00031341
**Ovary**	M	OvsY.up	RPS29	0.905888	0.00018685
**Ovary**	M	OvsY.up	RPS8	0.629402	0.00830688
**Ovary**	M	OvsY.up	SAMD4B	1.83227	0.00457234
**Ovary**	M	OvsY.up	SDC2	0.700234	0.04016952
**Ovary**	M	OvsY.up	SERP1	0.830625	0.03054197
**Ovary**	M	OvsY.up	SERPINB1	1.5428	0.00342572
**Ovary**	M	OvsY.up	SERTAD1	0.882494	0.00464793
**Ovary**	M	OvsY.up	SH3GLB1	0.854983	0.03812893
**Ovary**	M	OvsY.up	SIVA1	1.40094	0.02229872
**Ovary**	M	OvsY.up	SLC25A3	0.865038	0.00169989
**Ovary**	M	OvsY.up	SLC38A2	1.38711	0.00258813
**Ovary**	M	OvsY.up	SNU13	1.33939	0.00349024
**Ovary**	M	OvsY.up	SPAG9	3.14354	0.00136283
**Ovary**	M	OvsY.up	SPARCL1	0.968941	4.84E-05
**Ovary**	M	OvsY.up	SPART	0.626656	0.04618237
**Ovary**	M	OvsY.up	SPINT2	1.22148	0.01181223
**Ovary**	M	OvsY.up	SRPRA	2.44218	0.00919714
**Ovary**	M	OvsY.up	SRSF1	0.999032	0.00912985
**Ovary**	M	OvsY.up	SWAP70	1.46647	0.02053285
**Ovary**	M	OvsY.up	TBCA	1.33826	0.01121352
**Ovary**	M	OvsY.up	TBCB	1.43584	0.03489264
**Ovary**	M	OvsY.up	TDG	1.23922	0.02608692
**Ovary**	M	OvsY.up	THBS2	0.94607	0.00137354
**Ovary**	M	OvsY.up	TIMP1	0.888242	0.00173954
**Ovary**	M	OvsY.up	TMEM120A	1.80338	0.0389777
**Ovary**	M	OvsY.up	TMEM14A	1.17649	0.02388428
**Ovary**	M	OvsY.up	TMEM14B	0.712379	0.00196401
**Ovary**	M	OvsY.up	TMEM169	0.806019	0.03110226
**Ovary**	M	OvsY.up	TMEM176B	3.1122	0.00690821
**Ovary**	M	OvsY.up	TSC22D1	0.999942	0.0070494
**Ovary**	M	OvsY.up	TSPAN6	1.74028	0.00751025
**Ovary**	M	OvsY.up	TUBB3	1.05296	0.01619611
**Ovary**	M	OvsY.up	UBA1	0.774928	0.00470263
**Ovary**	M	OvsY.up	UBE2D2	1.20989	0.02012158
**Ovary**	M	OvsY.up	UBE2K	1.76081	0.0205189
**Ovary**	M	OvsY.down	UBE2L3	-2.04239	0.02370725
**Ovary**	M	OvsY.up	UGDH	0.631174	0.02254947
**Ovary**	M	OvsY.up	VCAN	1.57162	0.04306227
**Ovary**	M	OvsY.up	VEGFA	1.34029	0.00449882
**Ovary**	M	OvsY.up	VMP1	0.850501	0.02855451
**Ovary**	M	OvsY.up	VPS13C	1.34713	0.0492799

© 2023. Pei W et al. Published online at http://www.aginganddisease.org/EN/10.14336/AD.2023.0707-1

**Ovary**	M	OvsY.down	VPS29	-3.10794	0.01232905

Supplementary Table 3. DEGs of smooth muscle cells between the old and young NHP ovaries. Gene expression matrix along the pseudotime heatmap. Found at: https:// doi.org/10.7910/DVN/ D8RZKK.

Tissue	Cell Type	Class	Gene	Avg_logFC	P_val
**Ovary**	SMC	OvsY.up	ACTA2	0.678349	6.88E-06
**Ovary**	SMC	OvsY.down	AICDA	-1.57754	0.0009049
**Ovary**	SMC	OvsY.down	ANKRD35	-1.49226	0.02946943
**Ovary**	SMC	OvsY.up	APOD	1.21845	0.00024339
**Ovary**	SMC	OvsY.down	ARID5B	-0.50326	0.01555397
**Ovary**	SMC	OvsY.down	ATF4	-0.724	4.14E-06
**Ovary**	SMC	OvsY.up	ATP5A1	0.693943	0.04657936
**Ovary**	SMC	OvsY.down	ATP5G2	-0.586	0.00815078
**Ovary**	SMC	OvsY.up	ATP5I	0.860874	0.00211421
**Ovary**	SMC	OvsY.up	ATP6	1.15101	3.20E-09
**Ovary**	SMC	OvsY.up	B2M	0.967341	0.0145217
**Ovary**	SMC	OvsY.up	BTG2	0.703694	0.00018061
**Ovary**	SMC	OvsY.down	C7	-0.79072	2.00E-14
**Ovary**	SMC	OvsY.down	CCDC80	-1.10138	0.0346835
**Ovary**	SMC	OvsY.down	CD74	-1.1459	0.04999628
**Ovary**	SMC	OvsY.up	CHD2	0.741299	0.01357294
**Ovary**	SMC	OvsY.up	CNBP	0.664387	0.00258875
**Ovary**	SMC	OvsY.up	COX2	1.11173	7.07E-08
**Ovary**	SMC	OvsY.up	COX3	0.947244	1.08E-06
**Ovary**	SMC	OvsY.up	COX7B	0.803316	0.02672011
**Ovary**	SMC	OvsY.up	CRIP2	0.588661	0.00158476
**Ovary**	SMC	OvsY.up	CSRP1	0.584884	0.01780457
**Ovary**	SMC	OvsY.down	CST3	-1.36085	8.99E-05
**Ovary**	SMC	OvsY.down	DCN	-3.05192	6.55E-08
**Ovary**	SMC	OvsY.down	DDIT4	-1.98378	4.55E-07
**Ovary**	SMC	OvsY.up	EGFR	3.27833	1.67E-31
**Ovary**	SMC	OvsY.down	EIF3D	-1.16643	0.00139145
**Ovary**	SMC	OvsY.up	EIF4A1	0.923249	0.00109042
**Ovary**	SMC	OvsY.up	EIF4A3	0.781518	0.04878032
**Ovary**	SMC	OvsY.up	EIF4H	0.569801	0.0076308
**Ovary**	SMC	OvsY.down	ELF4	-1.81208	0.00086687
**Ovary**	SMC	OvsY.up	ELOB	0.794235	0.0231853
**Ovary**	SMC	OvsY.down	ENSMFAG00000001097	-0.5219	0.00637826
**Ovary**	SMC	OvsY.down	ENSMFAG00000001318	-0.75808	0.00156721
**Ovary**	SMC	OvsY.down	ENSMFAG00000001552	-0.80831	0.0414128
**Ovary**	SMC	OvsY.down	ENSMFAG00000002899	-0.55171	0.00058527
**Ovary**	SMC	OvsY.up	ENSMFAG00000015501	0.932351	0.00027197
**Ovary**	SMC	OvsY.down	ENSMFAG00000016983	-2.49498	4.72E-05
**Ovary**	SMC	OvsY.down	ENSMFAG00000021078	-1.89554	0.0032968
**Ovary**	SMC	OvsY.down	ENSMFAG00000021547	-0.69324	0.0076811
**Ovary**	SMC	OvsY.up	ENSMFAG00000026126	0.659182	0.00363251
**Ovary**	SMC	OvsY.up	ENSMFAG00000029971	0.939994	1.22E-05
**Ovary**	SMC	OvsY.down	ENSMFAG00000030186	-1.782	6.32E-15
**Ovary**	SMC	OvsY.up	ENSMFAG00000030267	0.504473	0.00367622
**Ovary**	SMC	OvsY.up	ENSMFAG00000030534	0.631055	0.00071722
**Ovary**	SMC	OvsY.up	ENSMFAG00000035941	1.03229	0.00149153
**Ovary**	SMC	OvsY.up	ENSMFAG00000036093_RHOA	0.745485	0.00092482
**Ovary**	SMC	OvsY.up	ENSMFAG00000036985	0.58104	0.00307063
**Ovary**	SMC	OvsY.down	ENSMFAG00000037443	-2.9195	7.30E-07
**Ovary**	SMC	OvsY.up	ENSMFAG00000038131	1.39231	2.33E-06
**Ovary**	SMC	OvsY.up	ENSMFAG00000039219	0.506021	0.01955355
**Ovary**	SMC	OvsY.up	ENSMFAG00000039395	1.38542	3.46E-07

**Ovary**	SMC	OvsY.up	ENSMFAG00000040760	0.850986	0.02883261
**Ovary**	SMC	OvsY.up	ENSMFAG00000043193_NDUFA4	0.766489	0.01741999
**Ovary**	SMC	OvsY.up	ENSMFAG00000045720	0.884084	0.01510664
**Ovary**	SMC	OvsY.down	FCGRT	-0.72903	0.01334557
**Ovary**	SMC	OvsY.down	GLUL	-0.70758	0.00010259
**Ovary**	SMC	OvsY.down	GRK6	-1.49687	0.01405835
**Ovary**	SMC	OvsY.up	GSTP1	0.677417	0.00341286
**Ovary**	SMC	OvsY.down	GTF2H1	-1.55438	0.00033076
**Ovary**	SMC	OvsY.down	GZMB	-1.41049	0.03265704
**Ovary**	SMC	OvsY.up	HMGN3	0.635188	0.01725802
**Ovary**	SMC	OvsY.down	HSP90AA1	-0.61118	0.01509599
**Ovary**	SMC	OvsY.down	HSPD1	-1.00065	0.00672231
**Ovary**	SMC	OvsY.down	IGFBP6	-2.45977	0.03447367
**Ovary**	SMC	OvsY.down	IGFBP7	-0.65104	0.00234881
**Ovary**	SMC	OvsY.up	IRF1	0.996931	0.00132716
**Ovary**	SMC	OvsY.up	ISYNA1	0.903091	0.00036036
**Ovary**	SMC	OvsY.up	JUNB	0.653412	0.01979956
**Ovary**	SMC	OvsY.up	KLF10	1.05606	1.11E-05
**Ovary**	SMC	OvsY.up	KRTCAP2	0.663798	0.01755475
**Ovary**	SMC	OvsY.down	LAPTM4A	-0.79696	0.00119439
**Ovary**	SMC	OvsY.down	Mafa-DRA	-1.25722	0.00264916
**Ovary**	SMC	OvsY.down	MDK	-0.88881	2.46E-06
**Ovary**	SMC	OvsY.up	MFGE8	0.796755	0.01184893
**Ovary**	SMC	OvsY.up	MGST3	0.609316	0.03364205
**Ovary**	SMC	OvsY.up	MRPL51	1.11047	0.00331256
**Ovary**	SMC	OvsY.up	MYC	0.680635	0.00118994
**Ovary**	SMC	OvsY.up	ND2	0.564061	0.00947173
**Ovary**	SMC	OvsY.up	ND3	3.04726	4.66E-19
**Ovary**	SMC	OvsY.up	ND4	1.01825	2.86E-05
**Ovary**	SMC	OvsY.up	NDUFA1	0.610248	0.0008077
**Ovary**	SMC	OvsY.up	NDUFA11	0.631398	0.02586826
**Ovary**	SMC	OvsY.up	NR4A1	0.827791	0.00797687
**Ovary**	SMC	OvsY.up	NR4A2	1.23009	0.00413498
**Ovary**	SMC	OvsY.down	OGN	-1.27057	2.26E-07
**Ovary**	SMC	OvsY.down	PALLD	-0.76882	0.00453869
**Ovary**	SMC	OvsY.up	PDLIM3	1.08656	1.17E-05
**Ovary**	SMC	OvsY.up	PFN1	0.841991	1.02E-05
**Ovary**	SMC	OvsY.down	PLA2G1B	-2.06076	0.00069938
**Ovary**	SMC	OvsY.down	PNRC1	-0.53501	0.02977418
**Ovary**	SMC	OvsY.down	PPP1R10	-0.60171	7.01E-05
**Ovary**	SMC	OvsY.up	PPP1R15A	0.547289	0.00632066
**Ovary**	SMC	OvsY.down	PSAP	-0.66287	0.04680705
**Ovary**	SMC	OvsY.down	PSMD3	-1.54224	0.00077869
**Ovary**	SMC	OvsY.down	RACK1	-0.67458	0.00157815
**Ovary**	SMC	OvsY.up	RAP1B	0.542375	0.02298961
**Ovary**	SMC	OvsY.up	RBM8A	1.02795	0.00470735
**Ovary**	SMC	OvsY.up	RBX1	1.06432	0.04527277
**Ovary**	SMC	OvsY.up	RGS16	1.87878	0.00214154
**Ovary**	SMC	OvsY.up	RHEB	0.774314	0.0047312
**Ovary**	SMC	OvsY.down	RPL28	-0.62883	0.00428551
**Ovary**	SMC	OvsY.down	RPL9	-0.55593	1.11E-05
**Ovary**	SMC	OvsY.down	RPS11	-0.56403	4.20E-05
**Ovary**	SMC	OvsY.down	RPS7	-0.80326	0.02571838
**Ovary**	SMC	OvsY.down	RPSA	-0.6185	0.01977225
**Ovary**	SMC	OvsY.down	RSRP1	-1.27897	3.43E-06
**Ovary**	SMC	OvsY.up	S100A4	1.13435	4.05E-06
**Ovary**	SMC	OvsY.up	SARAF	0.509079	0.04348822
**Ovary**	SMC	OvsY.down	SERPINE2	-2.15637	5.38E-05
**Ovary**	SMC	OvsY.down	SPARC	-1.02419	0.00944246

© 2023. Pei W et al. Published online at http://www.aginganddisease.org/EN/10.14336/AD.2023.0707-1

**Ovary**	SMC	OvsY.up	SQSTM1	0.825957	0.00185928
**Ovary**	SMC	OvsY.up	SRP14	0.6339	0.01038047
**Ovary**	SMC	OvsY.up	SUB1	0.964319	0.00193604
**Ovary**	SMC	OvsY.up	TAGLN2	1.1536	6.10E-05
**Ovary**	SMC	OvsY.down	TPT1	-0.61994	0.00262251
**Ovary**	SMC	OvsY.down	TXNIP	-1.34853	8.23E-15
**Ovary**	SMC	OvsY.up	VDAC2	0.710707	0.00733689
**Ovary**	SMC	OvsY.up	YWHAQ	0.56093	0.00175672

Supplementary Table 4. DEGs of stromal cells between the young and old NHP ovaries.

Tissue	Cell Type	Class	Gene	Avg_logFC	P_val
**Ovary**	SC	OvsY.down	AICDA	-0.80063	1.45E-05
**Ovary**	SC	OvsY.up	APOD	1.7504	1.17E-14
**Ovary**	SC	OvsY.up	APOE	0.874626	1.13E-09
**Ovary**	SC	OvsY.down	ATP5A1	-0.52271	0.04934536
**Ovary**	SC	OvsY.up	ATP5D	0.798107	1.43E-08
**Ovary**	SC	OvsY.up	BTG2	0.568507	7.30E-09
**Ovary**	SC	OvsY.down	CD74	-0.7567	1.15E-11
**Ovary**	SC	OvsY.up	CLU	1.15425	2.41E-07
**Ovary**	SC	OvsY.down	COX6A1	-0.52117	1.49E-06
**Ovary**	SC	OvsY.down	CST3	-1.03092	1.50E-10
**Ovary**	SC	OvsY.down	CTSK	-0.68885	1.20E-10
**Ovary**	SC	OvsY.down	DCN	-0.86659	8.03E-06
**Ovary**	SC	OvsY.up	EGFR	1.09867	7.69E-39
**Ovary**	SC	OvsY.up	EIF4A3	0.598141	0.0036785
**Ovary**	SC	OvsY.down	EIF5	-0.56433	0.00380578
**Ovary**	SC	OvsY.down	ENSMFAG00000001022	-0.58749	0.02502341
**Ovary**	SC	OvsY.down	ENSMFAG00000001143	-0.5842	0.00883344
**Ovary**	SC	OvsY.down	ENSMFAG00000001318	-0.56749	0.00044983
**Ovary**	SC	OvsY.up	ENSMFAG00000003168	0.572757	5.71E-08
**Ovary**	SC	OvsY.up	ENSMFAG00000007994	0.630161	1.72E-15
**Ovary**	SC	OvsY.up	ENSMFAG00000011451_RPL32	0.521913	1.89E-08
**Ovary**	SC	OvsY.up	ENSMFAG00000011609	3.32299	1.12E-30
**Ovary**	SC	OvsY.up	ENSMFAG00000012114	0.701585	1.11E-11
**Ovary**	SC	OvsY.up	ENSMFAG00000015501	0.722035	8.51E-12
**Ovary**	SC	OvsY.down	ENSMFAG00000021078	-1.37899	1.88E-05
**Ovary**	SC	OvsY.up	ENSMFAG00000027351	0.566119	6.44E-08
**Ovary**	SC	OvsY.up	ENSMFAG00000027717	0.60156	9.12E-10
**Ovary**	SC	OvsY.up	ENSMFAG00000030096	0.634781	9.07E-13
**Ovary**	SC	OvsY.up	ENSMFAG00000030598	0.750616	4.05E-21
**Ovary**	SC	OvsY.down	ENSMFAG00000030943	-0.64663	0.00022713
**Ovary**	SC	OvsY.down	ENSMFAG00000033843	-0.52627	0.00905864
**Ovary**	SC	OvsY.down	ENSMFAG00000035478	-0.581	0.00992812
**Ovary**	SC	OvsY.up	ENSMFAG00000043631	0.538052	1.24E-12
**Ovary**	SC	OvsY.down	ENSMFAG00000044097	-0.57563	1.38E-06
**Ovary**	SC	OvsY.up	ENSMFAG00000045720	0.747852	2.78E-13
**Ovary**	SC	OvsY.down	FBXO21	-0.53941	0.01053495
**Ovary**	SC	OvsY.down	GLUL	-1.42555	1.03E-11
**Ovary**	SC	OvsY.down	GNAS	-0.5744	8.13E-06
**Ovary**	SC	OvsY.down	GRK6	-1.55085	0.00634979
**Ovary**	SC	OvsY.up	GSTM5	1.05457	9.94E-09
**Ovary**	SC	OvsY.down	GZMB	-1.05983	0.00031619
**Ovary**	SC	OvsY.down	IDO2	-0.57109	0.00402219
**Ovary**	SC	OvsY.up	JUNB	0.547117	9.06E-09
**Ovary**	SC	OvsY.down	MDK	-0.66343	2.89E-06
**Ovary**	SC	OvsY.up	MT2	1.20194	6.57E-06
**Ovary**	SC	OvsY.up	MYC	0.728057	3.03E-11

© 2023. Pei W et al. Published online at http://www.aginganddisease.org/EN/10.14336/AD.2023.0707-1

**Ovary**	SC	OvsY.up	ND3	1.35182	2.05E-15
**Ovary**	SC	OvsY.down	NR2F2	-0.95278	6.05E-05
**Ovary**	SC	OvsY.down	PDK4	-1.03301	1.61E-13
**Ovary**	SC	OvsY.up	PRDX1	0.617236	0.00023654
**Ovary**	SC	OvsY.down	PSMD3	-1.13931	0.0024302
**Ovary**	SC	OvsY.down	RBM8A	-0.56304	0.00521863
**Ovary**	SC	OvsY.up	RBP1	0.50939	2.33E-07
**Ovary**	SC	OvsY.up	RPS15A	0.574898	4.23E-10
**Ovary**	SC	OvsY.down	SON	-0.61538	0.00597948
**Ovary**	SC	OvsY.down	SPARC	-1.20052	6.11E-13
**Ovary**	SC	OvsY.down	SPARCL1	-0.55893	1.76E-06
**Ovary**	SC	OvsY.down	SSR4	-0.59639	0.00647388
**Ovary**	SC	OvsY.down	TAGLN	-0.55453	0.00074739
**Ovary**	SC	OvsY.down	TSC22D3	-0.64507	1.00E-09
**Ovary**	SC	OvsY.down	TXNIP	-0.66529	1.50E-07
**Ovary**	SC	OvsY.down	WDR83OS	-0.61485	0.00153142
**Ovary**	SC	OvsY.down	YBX3	-0.5986	0.00098666

Supplementary Table 5. Antibodies used in this study.

Antibodies	Source	Identifier
**Rabbit anti-Decorin (DCN)**	Abcam	Cat# ab277636
**Rabbit anti-Apolipoprotein E (APOE)**	Abcam	Cat# ab183597
**Rabbit anti-SPARC**	Immunoway	Cat# YT5521
**Rabbit anti-SPARCL1**	Abcam	Cat# ab255597
**Mouse anti-Cystatin C (CST3)**	Immunoway	Cat# YM3626
**Rabbit anti-α-SMA (ACTA2)**	CST	Cat# 19245T
**Rabbit anti-PDLI3**	Immunoway	Cat# YT6467
**Rabbit anti-S100A4**	Abcam	Cat# ab197896
**Mouse anti-DNA/RNA damage (8-OHdG)**	Abcam	Cat# ab62623
**Rabbit anti-ZP1**	Immunoway	Cat# YT4994
**Rabbit anti-Histone H2A.X, phospho (Ser139) (γH2AX)**	CST	Cat# 9718
**Rabbit anti-ZP3**	Proteintech	Cat# 21279-1-AP
**Rabbit anti-CD3**	Abcam	Cat# ab16669
**Rabbit anti-CD68**	Servicebio	Cat# GB113150
**Mouse anti-CD8**	Servicebio	Cat# GB12068
**Mouse anti-Collagen I (COL1A1)**	Immunoway	Cat# YM3764
**Rabbit anti-MIS (AMH MIF)**	Abcam	Cat# ab229212
**Rabbit anti-CD45**	Servicebio	Cat# GB113885

© 2023. Pei W et al. Published online at http://www.aginganddisease.org/EN/10.14336/AD.2023.0707-1
